# Vitamin D & its analogues in type 2 diabetic nephropathy: a systematic review

**DOI:** 10.1186/s40200-015-0186-6

**Published:** 2015-07-15

**Authors:** Mrunalini K Chokhandre, Mahmoud I Mahmoud, Tahir Hakami, Mohammed Jafer, Aadil S Inamdar

**Affiliations:** Department of Clinical Pharmacology, Faculty of Medicine, Jazan University, Jazan, KSA; Department of Preventive Dental Sciences, College of Dentistry, Jazan University, Jazan, KSA

**Keywords:** 25 (OH) vitamin D, Kidney functions, Systematic review, Type 2 diabetic nephropathy, Vitamin D analogue

## Abstract

Nephropathy is one of the major complications of diabetes often leading to chronic kidney disease (CKD). Inflammation and oxidative stress are associated with pathogenesis of diabetic nephropathy (DN) and found to be regulated by nuclear receptors such as vitamin D receptors (VDR). Vitamin D and its analogues have been effectively used in patients with CKD. The review attempts to summarize the available evidence on the role of vitamin D in DN. Electronic databases (MEDLINE, EMBASE, and Cochrane Library) were searched for studies assessing the role of vitamin D or its analogues on kidney function in type 2 diabetic patients. Studies evaluating kidney functions (urinary albumin/protein creatinine ratio, albuminuria and eGFR) were included and quality and risk of bias assessment performed. Additionally effect on 25 (OH) vitamin D, calcium and HbA1c were evaluated. The mean or its % change along with their standard deviation (SD) was used for reporting our results. RevMan (V5.2) was used for data analysis. Six studies included in this review evaluated the role of cholecalciferol, calcitriol and paricalcitol in patients with DN. Study designs differed (three randomized, one non-randomized and two uncontrolled trials) with varying degree of quality and risk of biases. Vitamin D analogues showed significant improvement in kidney function in two randomized studies. None of the studies reported significant incidences of hypercalcemia. Vitamin D analogues show significant improvement of kidney function in DN. Randomized controlled trials with longer duration, comparing the efficacy of vitamin D and its analogues are needed.

**Electronic supplementary material:**

The online version of this article (doi:10.1186/s40200-015-0186-6) contains supplementary material, which is available to authorized users.

## Background

Diabetic nephropathy (DN) is a major microvascular complication of diabetes mellitus implicated in nearly 44 % of end stage renal disease (ESRD) patients that requires hemodialysis [1]. Type 2 diabetes has assumed a global pandemic proportion with estimates of approximately 382 million people with diabetes in 2013 and an expected rise to 592 million by 2035 with an anticipated increase in its complications such as nephropathy [2].

The pathogenesis of DN is multifactorial with contribution from several genetic and environmental factors. Diabetes induces various metabolic, biochemical and hemodynamic changes in kidneys. Major pathways leading to DN include: intracellular activation of polyol pathway and protein kinase C, advanced glycation end products (AGEs) and oxidative stress by reactive oxygen species, and glomerular hyperfiltration and hypertension leading to shear stress and mechanical stretch [3]. Hyperglycemia stimulates the production of Angiotensin II (Ang II), which exerts hemodynamic, inflammatory and profibrogenic effects on kidney cells [4]. Transcription factor like nuclear factor *κ* of activated B cells (NF-*κ*B) [5], pro-inflammatory cytokines such as tumor necrosis factor (TNF) and interleukin 1 (IL 1) [6], toll-like receptors 4 (TLR4) [7] , adiponectin [8] and nuclear hormone receptors [9] are related to inflammatory pathways in DN. Nuclear receptors are found to be the negative regulators of inflammation, oxidative stress and fibrosis. Vitamin D receptor (VDR) is one such nuclear receptor involved in various inflammatory pathways [10].

VDR is a transcription factor and intracellular receptor for 1-α,25-dihydroxyvitamin D3; an active metabolite of vitamin D. VDR has been found in proximal and distal tubular epithelium, glomerular parietal epithelium, collecting duct cells, juxtaglomerular apparatus and podocytes in kidneys [11]. Experimental studies have shown that VDR knock-out mice have higher expression of renin, angiotensin, and angiotensin receptors under diabetic conditions [12] and may develop severe renal injury resulting in early onset albuminuria, glomerulosclerosis and interstitial fibrosis mainly due to local RAAS activation [13]. Vitamin D exerts anti-inflammatory effects by modulating antigen presenting cells function [14], inhibiting dendritic cell maturation [15], reducing expression of cytokine interleukin-12 (IL-12), and repressing transcription of genes encoding interleukin 2 (IL2) and interferon gamma (INFγ) [16].

Several observational studies have confirmed this pathophysiologic link between Vitamin D deficiency and DN. The Third National Health and Nutrition Examination Survey (NHANES III) found an increase in the prevalence of albuminuria with decreasing 25(OH)D concentration [17]. Further evaluation in diabetic population of the survey showed independent association between Vitamin D deficiency and diabetic Nephropathy [18]. AusDiab, another large cohort study showed significant association between 25(OH)D deficiency and impaired eGFR [19]. A significant relationship between Vitamin D deficiency and nephropathy may exist, suggesting a possible role of Vitamin D in delaying the CKD progression.

Vitamin D and its analogues have been used in patients with CKD with considerable improvement in kidney functions, in terms of reduced urinary albumin creatinine ratio (UACR) and improved eGFR [20]. However studies evaluating the role of vitamin D in DN are few. There is a need to critically evaluate the available data and establish any beneficial association of the use of vitamin D or its analogues in DN. The aim of this review was thus to evaluate the efficacy of vitamin D and its analogues in the management of nephropathy in type 2 diabetic patients and to summarize the available evidence.

## Methods

### Studies

We conducted a search for all published, unpublished, ongoing observational or experimental studies, which evaluated the role of vitamin D or its analogues in the management of type 2 DN. All studies ‪of type 2 diabetic patients with nephropathy that reported at least one of the following outcome measures; Urinary Albumin Creatinine Ratio (UACR), Urinary Protein Creatinine Ratio (UPCR), Estimated Glomerular filtration Rate (eGFR) or albumin excretion rate were considered for inclusion. Studies with patients with diabetes other than Type 2, pregnant females, or patients with underlying debilitating conditions were excluded.

### Search methods

Electronic searches*:* Electronic databases [MEDLINE, EMBASE & the Cochrane Central Register of Controlled Trials (CENTRAL)] and other relevant web sites were comprehensively searched up to October 2014. The search was limited to human studies but not to language or area. The Medical Subject Headings (Mesh) keywords used to search databases included: “diabetes”, “nephropathy”, and “vitamin D”. Boolean operators were used to combine all searches (Additional file 1).

Other resources: Search was further supplemented by reviewing bibliographies of potentially relevant studies. Authors were contacted for any missing data from the studies included, or for potentially inclusive studies. Google Scholar search engine and database (Zetoc) were also searched for any relevant literature.

Titles, keywords and abstracts of all the relevant studies were examined by two authors (MKC and MIM) and articles potentially eligible for full text assessment were identified. Disagreements were resolved through discussion and consultation with a third reviewer (ASI).

### Selection of studies

Predetermined inclusion and exclusion criteria were used for all studies. Full text of all potentially relevant reports were obtained and reviewed by two authors (MKC and MIM). Study design, types of intervention (vitamin D and its analogue) and the different outcome measures (UACR, UPCR, eGFR) were among the inclusion criteria used in this review.

### Data extraction and Quality (risk of bias) assessment

For data extraction and to evaluate studies on quality, assessment sheets were specifically prepared for this review based on the Cochrane Handbook for Systematic Reviews of Interventions [21] and the Consolidated Standards of Reporting Trials (CONSORT) [22]. Assessment sheets addressed randomized and non-randomized studies. Two reviewers (MKC & ASI) made assessments of sequence generation, concealment allocation, blinding, incomplete reporting of outcome measurement data, while determining the internal validity of included studies. Two reviewers independently carried out data extraction (MKC & ASI). The means (or % change in the mean) and standard deviation (SD) of all included studies were extracted for all reported outcome measures. Characteristics of studies that were analyzed during data extraction were as follows: type of research design, study methods used, characteristics of participants included, details of outcome measure and type of analysis used.

### Data synthesis

Studies included in the review are presented according to the study design and then stratified according to the outcomes reported (UACR, UPCR, eGFR, albumin excretion rate). Additional outcomes evaluated include; serum 25 (OH) Vitamin D, serum calcium and glycosylated hemoglobin (HbA1C). Review Manager software (RevMan V5.2, 2012; The Nordic Cochrane Centre, Copenhagen, Denmark) was used for data analysis and preparing the review. Effect measures in this review were reported as Mean Difference (MD) or the percent change (%) in the mean differences, their 95 % confidence interval (CI) or their standard deviations SD [MD (CI/SD)]. The MD and SD were used for the visual representation of results in the forest plots. Data of the studies included was stratified based on the outcome measures reported and was to be pooled depending on the Chi^2^ and I^2^ test for heterogeneity in their results.

## Results

### Search results

**Fig. 1:**
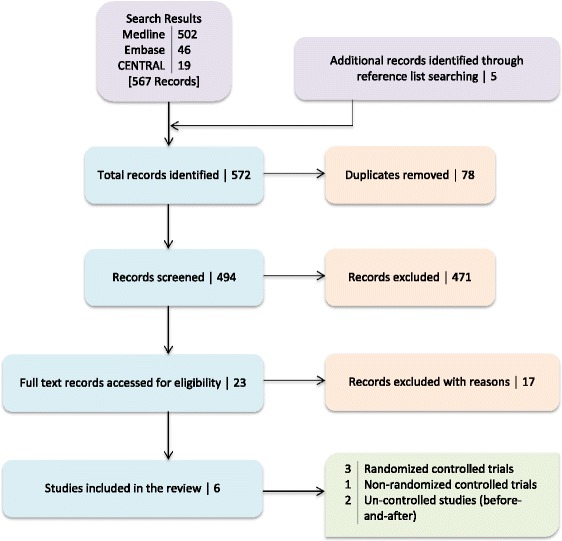
Study flow diagram

The search yielded a total of 572 papers (Medline-502, Embase-46, CENTRAL-19, others-5). After duplicates were removed and reports examined by title, keywords and abstract, they were screened for inclusion and exclusion. Twenty three papers were considered potentially eligible for inclusion and were retrieved for evaluation. Of these 23 papers, 17 were excluded after the evaluation (Fig. 1).

### Characteristics of studies

#### Included studies

Six studies were short listed for final analysis (Table 1). Three studies evaluated the effect of cholecalciferol (one randomized controlled trial (RCT) [23], one non-RCT [24] and one uncontrolled trial [25]), two of calcitriol (one RCT [26] and one uncontrolled trial [27]) and one RCT of paricalcitol [28] in patients with DN. The patients included in the studies were Vitamin D deficient with mild to moderate kidney dysfunction. Primary outcome measures were as follows: percent change UACR [28], change from baseline in UACR [23–25], change from baseline in UPCR [26] and albumin excretion rate (AER) [27]. Other efficacy measures evaluated in the studies included: eGFR [26, 28], blood pressure [23, 28] and Transforming Growth Factor-β (TGF-β) [25].

**Table 1:** Characteristics of included studies

Study ID (ref)	Design	Duration	Patients (n)	Baseline kidney function	Intervention	Outcome measures
Cholecalciferol						
Kim 2011 [25]	Uncontrolled, before-and-after study	4 months	63	(86 %) patients had eGFR < 60	Vitamin D deficiency: Cholecalciferol 40,000 units weekly for 8 weeks, and then same dose monthly, and Vitamin D insufficiency: Cholecalciferol 40,000 units monthly.	UACR, Transforming
						Growth Factor-β (TGF-β)
Huang 2012 [24]	Controlled, before-and-after study	6 months	46 (22 Vit D and 24 placebo)	UACR Mean (CI): Treated grp 97.4 (62.43,476.70) Untreated grp 114.4 (65.15,324.57)	Cholecalciferol daily at a dose of 800 IU/d over a 6-month period.	UACR
Nooshin 2013 [23]	Randomized double blind placebo controlled clinical trial	12 weeks	60 (30/30)	UACR mean (SD) 120.59 ± 145.40 95.49 ± 57.4	Oral vitamin D3 (pearl 50,000 IU) one pearl every week for 12 weeks	UACR
Calcitriol						
Bonakdaran 2012 [27]	Uncontrolled before-and-after study	8 weeks	43	Albumin excretion rate	0.5 μg calcitriol daily	Albumin excretion rate
				Mean (SD): 89.9 ± 192.4 GFR mean (SD): 107.2 ± 22.5		
Krairittichai 2012 [26]	Randomized clinical trial (open label)	16 weeks	91(46 vit D/45 placebo)	eGFR > 15	Oral calcitriol 0.5 mcg twice weekly or placebo	UPCR
Paricalcitol						
Zeeuw 2010 [28]	Randomized clinical trial (placebo-controlled, double-blind)	24 weeks	281	Microalbuminuria (28 %), macroalbuminuria (72 %)	1 μg paricalcitol (*n* = 93), or 2 μg paricalcitol (*n* = 95); placebo (*n* = 93)	Change in UACR, albuminuria, eGFR, Blood pressure

### Excluded studies

There were 17 studies excluded from this review. Fifteen of these excluded studies evaluated overall CKD patients but did not report any separate data for diabetic patients, while nine did not evaluate kidney functions as an outcome measure. One study evaluated the effect of vitamin D analogues with calcium on kidney functions and one RCT analyzing the effects of calcitriol on RAAS activation in type 2 DN patients is ongoing with no published results (Additional file 2).

### Quality assessment of included studies

Included studies showed high variations in overall quality. The randomized studies included in this review varied in their study design, structure and methodology. Overall risk of bias was low in two RCTs, however the risk of attrition bias was high as the studies failed to report patients follow up data [23, 28]. There was a high chance of performance bias in one RCT that did not report the method for randomization (sequence generation and allocation concealment) and blinding [26] (Table 2).

**Table 2:** Quality Assessment of Included Studies - Randomized Intervention studies

	External Validity	Internal validity									
Study ID (ref)			Performance			Detection		Attrition			Selection bias
	Sample^a^	Randomization	Randomization method	Allocation concealment	Participants blinded	Outcome measures, valid and reliable	Assessors blinded	Equal follow-up^b^	Attrition rate same for both groups?	Complete-ness^c^	groups similar at baseline
Krairittichai 2012 [26]	✔	✔	-	-	-	✔	-	✔	✔	✔	✔
Nooshin 2013 [23]	✔	✔	✔	-	✔	✔	✔	-	-	✔	✔
Zeeuw 2010 [28]	✔	✔	✔	✔	✔	✔	✔	-	-	✔	✔

*Notes*: A (✔) indicates the measure was adequately addressed in the study. The absence of a (✔) reflects that either the factor was not measured, or not reported, or did not meet the (✔) requirement; ^a^-studies received a (✔) if the sample was a random sample of included all eligible diabetic nephropathy with hypovitaminosis D patients from a defined area, or the sample size calculation was justified; ^b^-studies received a (✔) if groups were followed within 10 % of each other or for the same time; ^c^-studies received a (✔) if a full description of those lost-to-follow-up was not biased, or % of participants in the final analysis were ≥80

Non-randomized controlled trials had a weaker external validity as they lacked full reporting on sample size calculation and representativeness. Risk of performance bias was also high for the non-randomized studies. None of the studies reported blinding of the participant to the treatment [24, 25, 27]. There was a fair risk of detection bias in all the three non-randomized studies. Although the outcome measures were valid and reliable, none of these non-randomized studies reported blinding of the assessors to the outcomes. Risk of attrition and selection bias was high in one particular study with unequal follow up of the patient groups with no description of patients lost to follow up and participants in the final analysis being less than 80 % of the original [27]. None of these non-randomized studies identified any confounding factors or reported any adjustments in their statistical analysis [24, 25, 27] (Table 3).

**Table 3:** Quality Assessment of Included Studies – Non-Randomized Studies (NRS)

	External validity		Internal validity							
Study ID (ref)			Performance	Detection		Attrition			Selection bias	
	Sample^a^	Representative-ness^b^	Participants blinded	Outcome measures, valid and reliable	Assessors blinded	Equal follow-up^c^	Attrition rate same for both groups?	Complete-ness^d^	groups similar at baseline	Control of confounding
Kim 2011 [25]				✔				✔		
Huang 2012 [24]				✔		✔	✔	✔	✔	
Bonakdaran 2012 [27]				✔						

*Notes*: A (✔) indicates the measure was adequately addressed in the study. The absence of a (✔) reflects that either the factor was not measured, or not reported, or did not meet the (✔) requirement; ^a^-studies received a (✔) if the sample was a random sample of included all eligible diabetic nephropathy with hypovitaminosis D patients from a defined area, or the sample size calculation was justified; ^b^-studies received a (✔) if the % of participation was ≥80; ^d^-studies received a (✔) if groups were followed within 10 % of each other or for the same time; ^d^-studies received a (✔) if a full description of those lost-to-follow-up was not biased, or % of participants in the final analysis were ≥80; e-a (✔) indicates that there was adjustments done for confounders in the design or analysis

### Effect on renal function

#### UACR/UPCR/Albumin excretion rate

The RCT with cholecalciferol did not show any significant change in the UACR (*p* = 0.84). The treatment group had UACR (mean ± SD) of 120.59 ± 145.4 mg/g at baseline and 111.49 ± 128.99 mg/g at the end of treatment. The control group at baseline has UACR (mean ± SD) of 95.49 ± 57.4 mg/g while at end of treatment it was 88.43 ± 65.96 mg/g [23]. The uncontrolled study reported a significant change in UACR (mean; 95 % CI) with cholecalciferol from 12.7 (7.3–22.3) mg/mmol to 9.9 (5.5–17.9) mg/mmol (*p* = 0.0141) at 2 months. However, this change was not statistically significant at the end of 4 months [25]. The non-randomized study showed similar results as UACR (mean; 95 % CI) decreased significantly from 97.39 (62.43–476.70) mg/g to 71.65 (40.40–469.98) mg/g (*p* = 0.01) at 2 months, but again this change was not significant at 6 months [24].

**Fig. 2:**
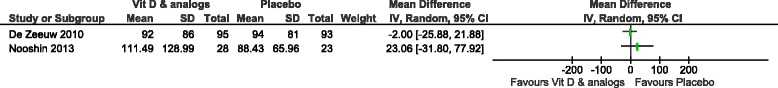
Forest Plot - Vit D & analogs in Kidney functions in DN - UACR

The RCT with calcitriol reported a significant difference in percent change in UPCR; −18.7 % in calcitriol group and +9.9 % in control group (*p* = 0.004) [26]. However, the uncontrolled study reported beneficial but non-significant change in albumin excretion rate with calcitriol treatment [27]. The RCT with paricalcitol showed a between-group difference versus placebo in the change in UACR of −18 % (95 %CI −32 to 0), (*p* = 0 · 053)]. The mean 24 h rate of albumin excretion also showed significant between-group difference of −28 % (95 %CI −43 to −8), (*p* = 0 · 009) [28]. Studies with available data on UACR were pooled together for a visual representation of the results (Fig. 2). Crude data was not available for one study for the forest plot [24]. There was very low heterogeneity between the studies (I^2^ = 0 %). Although the test for heterogeneity was not significant (Chi^2^ = 0.67, df = 1 [*p* = 0.41]), meta-analysis was considered inappropriate because of the small number of studies and use of different interventions (cholecalciferol and paricalcitol) [21].

#### eGFR

**Fig. 3:**
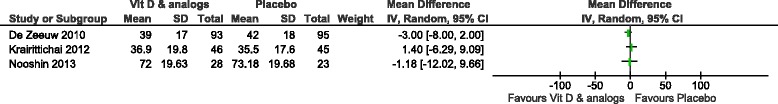
Forest Plot - Vit D & analogs in Kidney functions in DN - eGFR

Randomized trials with cholecalciferol [23], calcitriol [26] and paricalcitol [28] evaluated their effects on eGFR. Non-randomized trial of cholecalciferol [24] and uncontrolled studies of cholecalciferol [25] and calcitriol [27] did not report any crude data. None of these studies showed any significant benefit in terms of improvement in eGFR. Available data on studies reporting eGFR were also pooled for a forest plot (two did not report crude data [26, 28] (Fig. 3)). The test for heterogeneity was not significant (Chi^2^ = 0.67, df = 1 [*p* = 0.41]). There was no heterogeneity between the studies (I^2^ = 0 %). Even then, meta-analysis was still considered inappropriate with the small number of studies and use of different interventions (cholecalciferol, calcitriol and paricalcitol) [21].

### Effect on Serum 25 (OH) Vitamin D

The RCT with cholecalciferol showed a significant change from baseline in serum 25(OH)Vitamin D levels (mean ± SD) from 14.06 ± 7.76 to 71.23 ± 26.51 (*p* = 0.001) in the treatment group compared to 16.05 ± 6.08 to 17.63 ± 18.52 (*p* = 0.10) in the control group. The between-group difference in absolute percentage change was statistically significant (*p* < 0.0001) [23]. The non-randomized study also reported a significant change from baseline in serum 25(OH) Vitamin D levels at 6 months of treatment with cholecalciferol [17.56 (95 % CI 12.23, 23.83) ng/ml vs. 10.52 (95 % CI 7.75, 11.42) ng/ml]; *p* = 0.002 [24]. Likewise, a significant change in serum 25(OH) vitamin D levels (mean ± SD) from 15.6 ± 7 to 39.7 ± 12.8; *p* < 0.0001 was also seen at 4 months of treatment with cholecalciferol in the uncontrolled study [25]. Studies of vitamin D analogues (calcitriol and paricalcitol) did not report after treatment 25(OH) vitamin D levels.

### Effect on Serum calcium

The RCT of cholecalciferol reflected significant increases in levels of serum calcium (mean ± SD) from 9.22 ± 0.4 mg/dl to 9.75 ± 0.32 mg/dl (*p* < 0.0001) in the treatment group and from 9.40 ± 0.34 mg/dl to 9.76 ± 0.42 mg/dl (*p* < 0.002) in the control group. There was, however, no significant difference between the two groups (*p* = 0.14) [23]. The non-randomized trial of cholecalciferol showed no significant changes in the calcium levels within and between the two groups [24]. There were non-significant changes reported in the rest of the studies [25–28].

### Effect on HbA1c

The RCT evaluating the role of cholecalciferol reported a non-significant percentage change in HbA1c in both treatment and control group [23]. The non-randomized study [24] and the uncontrolled study [25] evaluating the effect of cholecalciferol on HbA1c also reported non-significant results. The RCT with calcitriol reported no significant change in HbA1c levels [26]. Interestingly, calcitriol effect on HbA1c was found to be statistically significant in the uncontrolled study where the percentage of HbA1c (mean ± SD) changed from 8.4 ± 1.8 to 7.6 ± 1.6 (*p* = 0.01) in the treatment group [27]. The paricalcitol study [28] did not report data on HbA1c.

## Discussion

Role of vitamin D and its analogues in the management of chronic kidney disease (CKD) has been addressed in meta-analyses that reports improvements in the biochemical parameters as well as reduced proteinuria without any significant adverse effects [20, 29]. The National Kidney Foundation-Kidney Disease Outcomes Quality Initiative (NKF-KDOQI) and Kidney Disease Improving Global Outcomes (KDIGO) clinical practice guidelines recommends Vitamin D supplementation in CKD patients with secondary hyperparathyroidism and associated mineral and bone disorders [30, 31]. However, literature on Vitamin D effect in the management of DN is limited. This systematic review summarizes the available evidence of use of vitamin D or its analogues in treatment of type 2 DN. We found six studies but with large variations in designs (three randomized controlled, one non-randomized controlled and two uncontrolled studies). Quality assessment revealed low risk of bias in two of the RCTs included in this review, while it was unclear-to-high in other studies. Pooling the data for meta-analysis, stratified according to the assigned intervention and based on the outcomes, was considered inappropriate due to differences in study designs and use of different interventions (cholecalciferol in three studies, calcitriol in two and paricalcitol in one study).

To our knowledge, this is the first systematic analysis of data available from studies evaluating the role of vitamin D or its analogues in the management of DN. The review showed that treatment with cholecalciferol can decrease UACR by 7.0 to 26.4 %. However, the studies included in the review found that the significant reduction in UACR seen during the initial two months of treatment was not sustained over four or six months [24, 25]. Proteinuria was also reduced significantly with the use of vitamin D analogues (paricalcitol and calcitriol) [26, 28]. However the change in proteinuria did not correspond to the change in eGFR. Vitamin D or its analogues did not show any deteriorating effects on kidney function. Similar observations were made by reviews and meta-analysis evaluating the effects of vitamin D supplementation in patients with CKD [20, 29].

Patients with DN in these studies had vitamin D deficiency, a common manifestation in patients with CKD [32, 33]. Clinical guidelines recommend a cholecalciferol dose of 1,000-2,000 IU/d for treatment of vitamin D deficiency in general population and a more aggressive dosing regimen in patients with CKD [34]. The chances of hypercalcemia with such doses are rare [35]. Findings from this review suggest that cholecalciferol supplementation can result in a significant dose-dependent elevation in the serum concentrations of 25(OH) D. The resulting increase in serum calcium concentration was however not significantly different from that in the placebo groups suggesting safety of high doses of cholecalciferol in DN patients. Studies evaluating the effect of vitamin D analogues in this review did not report the after treatment levels of 25-(OH) vitamin D or 1, 25- (OH) vitamin D and they did not report any significant increase in serum calcium concentration.

Another secondary efficacy end point evaluated in this review was HbA1c. Observational studies have established a causal relationship between vitamin D deficiency and diabetes [36]. Findings in this review suggest that cholecalciferol and paricalcitol do not affect HbA1c but calcitriol may reduce it. One meta-analysis reported a small beneficial improvement in fasting blood glucose and insulin resistance in patients with abnormal glucose tolerance receiving vitamin D but no change in HbA1c was reported. In patients with normal fasting glucose levels no improvement was seen in any outcome measures [37]. Another recent review also reported no significant improvement in glycemic parameters with vitamin D supplementation in patients with diabetes [38].

The role of various proinflammatory molecules have been identified in the pathogenesis of diabetic nephropathy [39]. Vitamin D exhibits anti-inflammatory properties by modulating such proinflammatory molecules [14–16]. Clinical data also suggest a possible anti-inflammatory role of calcitriol in DN. Low serum 25(OH) D3 levels were found to be associated with increased serum and urinary markers of inflammation such as TNF-α, IL-6, and ICAM-1 which decreased significantly after calcitriol treatment [40]. One of the studies included in this review evaluated the effect of cholecalciferol supplementation on inflammatory markers such as urinary monocyte chemoattractant protein-1 (MCP-1) and transforming growth factor-β1 (TGF- β1). This study reported a significant decrease in urinary TGF- β1 suggesting a potential beneficial effect of vitamin D [25].

The potential role of vitamin D deficiency in cardiovascular morbidity and mortality has been summarized earlier [41, 42]. Hypovitaminosis D is associated with asymptomatic cardiovascular disease (CVD) in type 2 diabetic patients with nephropathy [43]. Moreover, vitamin D deficiency was also found to be prevalent in hemodialysis patients and is associated with higher all-cause early mortality [44]. The evidence supporting the role of vitamin D supplementation in such conditions is lacking. One study with Alphacalcidiol showed survival advantage in chronic HD patients [45]. However, a recent meta-analysis concludes that the clinical evidence on effect of vitamin D supplementation on cardiovascular mortality is insufficient [46]. None of the studies included in this review addressed the risk of CVD or survival in type 2 DN patients.

Studies included in this review were grouped according to the study design and sub grouped according to the different outcomes. With variations in the study design, interventions and outcomes; pooling of the results for meta-analysis was considered inappropriate. Methodology, conduct and reporting of this review were based on the recommended guidelines [21]. There is a general criticism about a one-size-fit-all approach with systematic reviews. This review however uses an adapted risk of bias and quality assessment tool. Even though, certain limitations of this review have to be acknowledged. The search strategy was comprehensive, but there are still chances of studies being missed. Authors were contacted, but were unable to provide us with the unpublished or missing data. Although all reviewers contributed to assessment of quality of the studies included, there is still a fair likelihood of personal bias in the review.

## Conclusions

The six studies included in this review evaluating the role of vitamin D or its analogues in type 2 diabetic nephropathy have large variations in their study designs. Vitamin D analogues showed significant beneficial effects on kidney functions. Results also indicated benefits with high dose cholecalciferol early in the treatment however the changes were not sustained. Results of these short duration studies may be ambiguous and should be read with caution. Vitamin D or its analogues do not have any negative influence on kidney functions but monitoring serum calcium levels is important to ascertain the safety in patients with DN. Experimental before-and-after studies and studies without control or comparison groups may not be a suitable study design for evaluating the effects of vitamin D. Studies with standardized dose and duration of treatment of vitamin D or its analogue would provide a clearer picture on the effects of this supplementation in DN. Randomized double-blinded controlled trials are needed to further refine these findings and strengthen the current evidence on the effects of Vitamin D and its analogues on diabetic nephropathy.

## Additional files

Additional file 1:
**Search Strategy used in the review.**
Additional file 2:
**Table listing studies excluded from the review, with reasons why they were excluded.**
