# Supplementary material for: Vitamin D & its analogues in type 2 diabetic nephropathy: a systematic review
Source: J Diabetes Metab Disord. 2015 Jul 15;14:58. doi: 10.1186/s40200-015-0186-6 (PMC4502529; doi:10.1186/s40200-015-0186-6)
Supplement: Additional file 1: — Search Strategy used in the review. [file 40200_2015_186_MOESM1_ESM.pdf]

Supplementary Data 1

MEDLINE Search Strategy

#1

(("vitamin d"[MeSH Terms] OR "vitamin d"[All Fields] OR "ergocalciferols"[MeSH Terms] OR "ergocalciferols"[All Fields])

#2

("diabetes mellitus"[MeSH Terms] OR ("diabetes"[All Fields] AND "mellitus"[All Fields]) OR "diabetes mellitus"[All Fields] OR "diabetes"[All Fields]))

#3

("kidney diseases"[MeSH Terms] OR ("kidney"[All Fields] AND "diseases"[All Fields]) OR "kidney diseases"[All Fields] OR "nephropathy"[All Fields])

#4

(#1 AND #2 AND #3)

532 RESULTS
