# Supplementary material for: Vitamin D & its analogues in type 2 diabetic nephropathy: a systematic review
Source: J Diabetes Metab Disord. 2015 Jul 15;14:58. doi: 10.1186/s40200-015-0186-6 (PMC4502529; doi:10.1186/s40200-015-0186-6)
Supplement: Additional file 2: — Table listing studies excluded from the review, with reasons why they were excluded. [file 40200_2015_186_MOESM2_ESM.pdf]

Supplementary Table 1. Excluded Studies & Reasons for Exclusion

Sr No	Study ID	Reason for exclusion
1	Aperis et al 2011 (1)	No separate data for diabetic patients
2	Basturk et al 2011 (2)	No separate data for diabetic patients; kidney function not evaluated
3	Blair et al 2008 (3)	No separate data for diabetic patients; kidney function not evaluated
4	Coronel et al 2011 (4)	No separate data for diabetic patients
5	Hervas et al 2011 (5)	No separate data for diabetic patients
6	Nicola el al 2012 (6)	No separate data for diabetic patients
7	Ogawa et al 2012 (7)	No separate data for diabetic patients; kidney function not evaluated (survival)
8	Miller et al 2012 (8)	No separate data for diabetic patients; kidney function not evaluated (survival)
9	Agarwal et al 2005 (9)	No separate data for diabetic patients
10	Sugiura et al 2010 (10)	No separate data for diabetic patients; kidney function not evaluated (survival)
12	Nathan et al 2013 (11)	No separate data for diabetic patients; kidney function not evaluated
13	Cianciolo et al 2013 (12)	No separate data for diabetic patients; kidney function not evaluated
14	Floege et al 2010 (13)	No separate data for diabetic patients; kidney function not evaluated
15	Jean et al 2008 (14)	No separate data for diabetic patients; kidney function not evaluated
16	Mustafar et al 2014 (15)	Vitamin D analogues and calcium effect evaluated in CKD patients
17	Brown et al 2013 (16)	Ongoing, results not yet available

References:

1. Aperis G, Paliouras C, Zervos A, Arvanitis A, Alivanis P. The role of paricalcitol on proteinuria. J Ren Care. 2011 Jun;37(2):80–4.

2. Basturk T, Unsal A, Ulas T. Effect of cholecalciferol on parathyroid hormone and vitamin D levels in chronic kidney disease. Minerva Urol E Nefrol Ital J Urol Nephrol. 2011 Dec;63(4):287–92.

3. Blair D, Byham-Gray L, Lewis E, McCaffrey S. Prevalence of vitamin D [25(OH)D] deficiency and effects of supplementation with ergocalciferol (vitamin D2) in stage 5 chronic kidney disease patients. J Ren Nutr Off J Counc Ren Nutr Natl Kidney Found. 2008 Jul;18(4):375–82.

4. Coronel F, Rodríguez-Cubillo B, Cigarrán S, Gomis A. Effects of oral paricalcitol on hyperparathyroidism and proteinuria in peritoneal dialysis patients. Adv Perit Dial Conf Perit Dial. 2011;27:130–3.

5. Hervás Sánchez JG, Prados Garrido MD, Polo Moyano A, Cerezo Morales S. Effectiveness of treatment with oral paricalcitol in patients with pre-dialysis chronic kidney disease. Nefrol Publ Of Soc Esp Nefrol. 2011;31(6):697–706.

6. Nicola LD, Conte G, Russo D, Gorini A, Minutolo R. Antiproteinuric effect of add-on paricalcitol in CKD patients under maximal tolerated inhibition of renin-angiotensin system: a prospective observational study. BMC Nephrol. 2012 Nov 20;13(1):150.

7. Ogawa M, Ogawa T, Inoue T, Otsuka K, Nitta K. Effect of alfacalcidol therapy on the survival of chronic hemodialysis patients. Ther Apher Dial Off Peer-Rev J Int Soc Apher Jpn Soc Apher Jpn Soc Dial Ther. 2012 Jun;16(3):248–53.

8. Miller JE, Molnar MZ, Kovesdy CP, Zaritsky JJ, Streja E, Salusky I, et al. Administered paricalcitol dose and survival in hemodialysis patients: a marginal structural model analysis. Pharmacoepidemiol Drug Saf. 2012 Nov;21(11):1232–9.

9. Agarwal R, Acharya M, Tian J, Hippensteel RL, Melnick JZ, Qiu P, et al. Antiproteinuric effect of oral paricalcitol in chronic kidney disease. Kidney Int. 2005 Dec;68(6):2823–8.

10. Sugiura S, Inaguma D, Kitagawa A, Murata M, Kamimura Y, Sendo S, et al. Administration of alfacalcidol for patients with predialysis chronic kidney disease may reduce cardiovascular disease events. Clin Exp Nephrol. 2010 Feb;14(1):43–50.

11. Hewitt NA, O'Connor AA, O'Shaughnessy DV, Elder GJ. Effects of Cholecalciferol on Functional, Biochemical, Vascular, and Quality of Life Outcomes in Hemodialysis Patients. Clin J Am Soc Nephrol [Internet]. 2013 Jul 3 [cited 2014 Dec 9];8(7):1143–9. Available from: http://cjasn.asnjournals.org/content/8/7/1143

12. Cianciolo G, La Manna G, Della Bella E, Cappuccilli ML, Angelini ML, Dormi A, et al. Effect of vitamin D receptor activator therapy on vitamin D receptor and osteocalcin expression in circulating endothelial progenitor cells of hemodialysis patients. Blood Purif. 2013;35(1-3):187–95.

13. Floege J, Raggi P, Block GA, Torres PU, Csiky B, Naso A, et al. Study design and subject baseline characteristics in the ADVANCE Study: effects of cinacalcet on vascular calcification in haemodialysis patients. Nephrol Dial Transplant Off Publ Eur Dial Transpl Assoc - Eur Ren Assoc. 2010 Jun;25(6):1916–23.

14. Jean G, Terrat JC, Vanel T, Hurot JM, Lorriaux C, Mayor B, et al. Evidence for persistent vitamin D 1-alpha-hydroxylation in hemodialysis patients: evolution of serum 1,25-dihydroxycholecalciferol after 6 months of 25-hydroxycholecalciferol treatment. Nephron Clin Pract. 2008;110(1):c58–65.

15. Mustafar R, Mohd R, Ahmad Miswan N, Cader R, Gafor HA, Mohamad M, et al. The effect of calcium with or without calcitriol supplementation on renal function in patients with hypovitaminosis d and chronic kidney disease. Nephro-Urol Mon. 2014 Jan;6(1):e13381.

16. Brown JM, Secinaro K, Williams JS, Vaidya A. Evaluating hormonal mechanisms of vitamin D receptor agonist therapy in diabetic kidney disease: the VALIDATE-D study. BMC Endocr Disord. 2013 Aug 23;13:33.
